# Retraction: Comparative evaluation of the incidence of postoperative pulmonary complications after minimally invasive valve surgery vs. full sternotomy: a systematic review and meta-analysis of randomized controlled trials and propensity score-matched studies

**DOI:** 10.3389/fcvm.2024.1422760

**Published:** 2024-05-01

**Authors:** Frontiers Editorial Office

A Retraction of the Systematic Review Article Comparative evaluation of the incidence of postoperative pulmonary complications after minimally invasive valve surgery vs. full sternotomy: a systematic review and meta-analysis of randomized controlled trials and propensity score-matched studies By Mohamed MA, Ding S, Ali Shah SZ, Li R, Dirie NI, Cheng C and Wei X (2021). Front. Cardiovasc. Med. 8:724178. doi: 10.3389/fcvm.2021.724178

Following publication, concerns were raised regarding the scientific validity of the article. An investigation was conducted in accordance with Frontiers’ policies. The authors failed to provide a satisfactory explanation and as a result, the conclusions of the article have been deemed unreliable and the article is retracted.

This retraction was approved by the Chief Editors of Cardiovascular Medicine and the Chief Executive Editor of Frontiers. The authors agree to this retraction.

